# Intelligent Detection Method of Atrial Fibrillation by CEPNCC-BiLSTM Based on Long-Term Photoplethysmography Data

**DOI:** 10.3390/s24165243

**Published:** 2024-08-14

**Authors:** Zhifeng Wang, Jinwei Fan, Yi Dai, Huannan Zheng, Peizhou Wang, Haichu Chen, Zetao Wu

**Affiliations:** 1 School of Mechatronics Engineering and Automation, Foshan University, Foshan 528000, China; zhifengw0816@163.com (Z.W.); jinweifan98@163.com (J.F.); hnzheng@foxmail.com (H.Z.); zetaowu0818@163.com (Z.W.); 2 Guangdong Provincial Key Laboratory of Industrial Intelligent Inspection Technology, Foshan University, Foshan 528000, China; 3 School of Education, City University of Macau, Macau 999078, China; 4 Cosmetic Dermatology Department, Dermatology Hospital of Southern Medical University, Guangzhou 510091, China; zxwangpeizhou@student.pumc.edu.cn

**Keywords:** atrial fibrillation, photoplethysmography, long-term, CEPNCC-BiLSTM, *ET-score*

## Abstract

Atrial fibrillation (AF) is the most prevalent arrhythmia characterized by intermittent and asymptomatic episodes. However, traditional detection methods often fail to capture the sporadic and intricate nature of AF, resulting in an increased risk of false-positive diagnoses. To address these challenges, this study proposes an intelligent AF detection and diagnosis method that integrates Complementary Ensemble Empirical Mode Decomposition, Power-Normalized Cepstral Coefficients, Bi-directional Long Short-term Memory (CEPNCC-BiLSTM), and photoelectric volumetric pulse wave technology to enhance accuracy in detecting AF. Compared to other approaches, the proposed method demonstrates faster preprocessing efficiency and higher sensitivity in detecting AF while effectively filtering out false alarms from photoplethysmography (PPG) recordings of non-AF patients. Considering the limitations of conventional AF detection evaluation systems that lack a comprehensive assessment of efficiency and accuracy, this study proposes the *ET-score* evaluation system based on F-measurement, which incorporates both computational speed and accuracy to provide a holistic assessment of overall performance. Evaluated with the *ET-score*, the CEPNCC-BiLSTM method outperforms EEMD-based improved Power-Normalized Cepstral Coefficients and Bi-directional Long Short-term Memory (EPNCC-BiLSTM), Support Vector Machine (SVM), EPNCC-SVM, and CEPNCC-SVM methods. Notably, this approach achieves an outstanding accuracy rate of up to 99.2% while processing PPG recordings within 5 s, highlighting its potential for long-term AF monitoring.

## 1. Introduction

Stroke is a leading cause of death and disability worldwide [1,2]. Reliable research has established a strong correlation between AF and ischemic stroke [3]. Furthermore, strokes associated with AF carry higher risks [4,5,6,7,8,9]. Although there are numerous methods for detecting AF based on electrocardiogram (ECG) and ECG-like signals, these methods have inherent limitations [10]. While ECG serves as the gold standard for AF detection and accurately analyzes heart rate and rhythm [11], identifying paroxysmal AF with short-period ECG recordings remains challenging due to its paroxysmal and complex nature [12]. Additionally, long-term ECG monitoring requires medical professionals, disrupts daily activities, and increases monitoring costs. Therefore, there is an urgent need to develop a novel, accurate, and efficient method for processing long-term PPG recordings that overcomes the limitations of traditional methods while enhancing AF detection rates.

Studies suggest that PPG recordings hold potential as an alternative to ECG for AF detection. Chong et al. [13] proposed that PPG waveforms may contain features beyond heart rate. However, further validation is required regarding the accuracy of PPG devices and the ability of physicians to detect AF based on PPG recordings [14]. To address these challenges, researchers have explored the application of image processing techniques and artificial intelligence methods in analyzing PPG recordings [15,16,17]. With advancements in computing technology, deep learning methods have gained significant attention [18]. For instance, Wu et al. [19] utilized a smartwatch device to obtain 30 s PPG recordings and achieved an overall accuracy of 92.38% using a hybrid deep learning model called Res-BiANet across 28,440 signal segments from 102 patients. Although studies have demonstrated the effective detection of AF using shorter periods of PPG recording in some cases, the paroxysmal nature of AF presents the risk of missed detection with short-duration signal segments [20].

PPG devices have attracted significant attention due to their potential for long-term monitoring and affordability [21]. However, the application of long-term PPG recordings is limited as it increases the computation time and poses challenges for rapid AF detection. Chen et al. [22] developed a novel smart bracelet that utilizes long-term PPG recordings to detect AF, demonstrating the integration potential of PPG with smart devices. For instance, Saarinen et al. [23] employed 5 min PPG and ECG recordings to analyze AF through a random forest algorithm, achieving an accuracy of 97.4%. Similarly, Kotlyarov et al. [24] analyzed over 2 min PPG recordings using a support vector machine (SVM) with a processing time of approximately 3 min, achieving sensitivity of 92.3%, specificity of 94.7%, and accuracy of 93.5%. While utilizing long-term PPG recordings can indeed enhance detection performance, the extended processing time is unfavorable for rapid AF diagnosis [25]. Moreover, there is currently no established standard in the field of AF diagnosis regarding the selection criteria for term length and processing time when utilizing PPG recordings. Evaluating the efficiency of an AF diagnostic method requires considering the appropriate recording duration, along with corresponding signal processing and classification techniques.

In the field of AF detection, convolutional neural networks (CNNs) are widely adopted due to their excellent spatial feature extraction capabilities [26]. However, they face challenges in capturing temporal dependencies within electrophysiological signals. To address this issue, recurrent neural networks (RNNs) have been introduced. For example, Senturk et al. [27] integrated ECG and PPG recordings as inputs into an RNN with the objective of estimating continuous blood pressure. Xu-K et al. [28] employed RNNs to achieve precise PPG cardiogram segmentation, resulting in three key indicators for heart rate variability estimation. However, traditional RNNs often face the issue of gradient vanishing when processing long-sequence data. To address this limitation, the bidirectional long short-term memory (BiLSTM) model has been employed [29], with the aim of enhancing the model’s capacity to capture contextual information in time series analysis and thereby improving AF event recognition.

In conclusion, this paper proposes an innovative method named CEPNCC-BiLSTM for the precise classification of AF characteristics based on long-term PPG recordings. To address the challenges in AF detection from PPG recordings and improve the processing speed of long-term PPG data, the proposed method enhances power normalized cepstral coefficients (PNCC) by incorporating complementary ensemble empirical mode decomposition (CEEMD) to extract frequency domain features. These features are then combined with time domain features to form a feature matrix. Subsequently, the feature matrix is fed into a BiLSTM neural network for accurate and effective identification and classification of AF characteristics. Additionally, to address the limitations of traditional evaluation systems that assess efficiency and accuracy comprehensively, this paper introduces a novel evaluation method called the *ET-score*, which incorporates time factors into the F-measure. This enables a comprehensive evaluation of various methods’ efficiency and accuracy.

## 2. Materials and Methods

### 2.1. Data

In this study, the effectiveness of the proposed algorithm is validated through experimental analysis. The PPG recordings are sourced from the MIT-BIH-MIMIC-III database at the Massachusetts Institute of Technology (MIT). MIMIC-III (‘Medical Information Mart for Intensive Care’) is an internationally recognized ECG database that accurately represents differences in pulse classification algorithms. The MIMIC-III Waveform Database is a large single-center database comprising vital signs, medications, laboratory measurements, observations, etc. [30]. In this research, PPG recordings were collected from both patients with AF and individuals exhibiting normal sinus rhythm (NSR) in the Synthetic Dataset [31] (1500 records in total). Among them, the signals of AF patients were confirmed by the diagnostic opinions provided by MIMIC-III. Furthermore, to validate the ability of the proposed method to detect AF patients with additional diseases, a small number of AF patients with other diseases were also included in the dataset.

To develop the classification models, the dataset was divided into two parts: a training set and a test set. The former was used for model training and the latter was kept as unseen data to test the classification performance. To ensure similar rhythm distributions in both datasets, 66.6% of the patients were assigned to the training set and 33.3% were assigned to the test set based on the rhythm. The details of the rhythm distributions are presented in Table 1, including the percentage of beats in each class and the number of patients having that rhythm.

### 2.2. Evaluation Indicators

To evaluate the sensitivity and time complexity of different methods simultaneously and provide a more intuitive assessment of their overall performance, this paper introduces the *ET-score* to evaluate the performance of AF detection methods using the following evaluation formula:

(1) $${ET}_{score}=\frac{\left(\beta^{2}+1\right)\times {(F}_{AF}\times F_{NSR})}{\beta^{2}\cdot (F_{AF}+F_{NSR})}\times time,$$

where β represents the estimated parameter, and in this paper, β is set to 1. $F_{AF}$ and $F_{NSR}$ are the F-measures corresponding to AF and NSR, respectively. The calculation speed factor of the AF detection method is time, whose maximum value is 100 and decreases by 2 in order of calculation speed ranking.

Several evaluation methods have been investigated to assess the accuracy and computation time of AF detection algorithms, aiming to demonstrate their performance in different aspects. However, none of these methods can comprehensively evaluate the overall performance of the algorithm. When comparing multiple methods, the *ET-score* incorporates a time factor related to the computational speed in addition to the accuracy rate. The *ET-score* considers both efficiency and accuracy, providing an assessment of the AF detection methods’ effectiveness. A high *ET-score* indicates that a method is both accurate and computationally efficient.

### 2.3. Preprocessing and Signal Analysis

**Figure 1:**
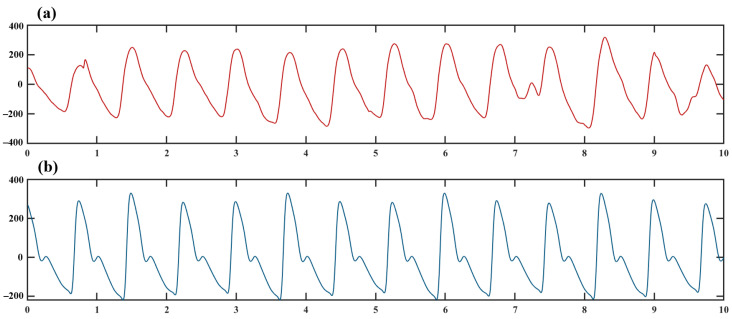
10 s PPG recordings after Preprocessing (**a**) AF Patient (**b**) Sinus rhythm.

The preprocessed PPG recordings commence from a peak and have a duration of 2 min. Examples of 10 s segments of the PPG waveforms for different rhythm types are presented in Figure 1. Compared to NSR, AF patients exhibit intermittent fluctuations in PPG recordings, which are significant factors influencing AF detection [32]. To determine the optimal PPG feature extraction times for AF identification, PPG recordings were divided into 1 min, 2 min, and 5 min for pre-experiments. The feature extraction time, network training time, classification accuracy, and *ET-score* were calculated, respectively. Our study reveals that 2 min PPG recordings achieve the highest *ET-score*; in other words, 2 min PPG recordings strike a balance between efficiency and convenience, as shown in Table 2. Tang et al. [33] also demonstrated that longer signals result in better classification performance, but excessively long signal records will prolong the computation time. Based on the results of the pre-experiments, PPG recordings with a length of 2 min containing 15,000 data points were utilized in this study. The selection of different signals was performed by detecting local maxima, and all the signals were normalized to ensure the accuracy of the subsequent algorithm.

### 2.4. CEPNCC Algorithm Details

PNCC is a speech-processing method that demonstrates strong efficiency in dealing with physiological signals [34]. Figure 1 shows the statistical characteristics of the PPG recordings varying with time, indicating that it is a non-smooth signal. However, the application of the fast Fourier transform (FFT) for signal preprocessing proves to be ineffective for non-smooth signals. Chen et al. [35] proposed an EEMD-based improved Power-Normalized Cepstral Coefficients (EPNCC) approach by employing Ensemble Empirical Mode Decomposition (EEMD) for signal preprocessing instead of FFT. However, EEMD requires hundreds of calculations to reduce the residual error to a low level. In order to enhance efficiency while reducing error, the CEEMD [36] and EEMD were utilized to preprocess PPG recordings, with their respective processing times detailed in Table 3. Notably, CEEMD exhibits a significant advantage in terms of processing time.

CEEMD is used to preprocess the PPG recordings with the following steps.

Step 1: Let N be the overall number of processing of the original signal, indicating the total number of white noise signals added.

Step 2: A positive white noise signal sequence $\left\{n_{i}\right\}$ and a negative white noise signal sequence $\left\{-n_{i}\right\}$ are added to the original signal s(t), forming two new sets of signals, ${r_{i}}^{+}(t)$ and ${r_{i}}^{-}(t)$, respectively, where i=1, 2, …, N.

Step 3: Using EMD to decompose ${r_{i}}^{+}(t)$ and ${r_{i}}^{-}(t)$ separately, a series of intrinsic modal functions (IMFs) are obtained. Note that $I_{ij}^{+}(t)$ and $I_{ij}^{-}(t)$ are the jth intrinsic modal functions of ${r_{i}}^{+}(t)$ and ${r_{i}}^{-}(t)$, respectively.

Step 4: Let $I_{j}(t)$ be the *j*th intrinsic modal function of the reconstructed signal where $I_{j}(t)$ is the average of the components $I_{ij}^{+}(t)$ and $I_{ij}^{-}(t)$, as shown in the following equation:

(2) $$I_{j}\left(t\right)=\frac{1}{2N}\sum_{i=1}^{N}\left(I_{ij}^{+}\left(t\right)+I_{ij}^{-}(t)\right).$$

Step 5: Let p be the total number of all IMFs in the reconstructed signal and let r(t) be the residual of the reconstructed signal. The corresponding IMFs are averaged as the result of the decomposition, as shown in the following equation:

(3) $$s\left(t\right)=\sum_{j=1}^{p}I_{j}^{+}\left(t\right)+r\left(t\right).$$

After decomposing the PPG recordings using CEEMD, each IMF component and the residuals were obtained. Due to the time-varying and random nature of the noise, the IMF components after EEMD decomposition will vary. The large differences in these IMF components are meaningless for characterizing the impulse signal properties. Therefore, before extracting PNCC frequency domain features, a correlation analysis was conducted on the EEMD-decomposed IMF components to filter out those that characterize the impulse signal. Correlation analysis can determine the coherence between each IMF component and the original PPG recordings, and the expression for the coherence coefficient is

(4) $$\mu_{i}=\frac{cov({imf}_{i},\;Sig)}{\sqrt{Var\left[{imf}_{i}\right]\;Var[Sig]}},$$

where *n* is the total number of the obtained IMFs, Sig is the original PPG record, and the desired IMF is selected by the difference from the set threshold value.

After the PPG record is processed by EEMD, the components are arranged and expressed as a feature matrix composed of IMFs. Here are the CEPNCC feature extraction steps:

Step 1: The estimated power spectrum $P_{i}\left(\omega \right)$ for each IMF is calculated as follows:

(5) $$P_{i}\left(\omega \right)=\underset{T\to \infty }{\lim}\frac{\left|{IMF}_{i}\right|}{2\pi T}$$

Step 2: The power estimate$\;P_{i}\left(\omega \right)$ is input into the Gammatone filter for filtering, and the time domain impulse response of the Gammatone filter is formulated as follows:

(6) $$G\left(t\right)=at^{n-1}e^{-2\pi wt}\cos\left(2\pi f_{0}t+\varphi \right),\left(t>0\right)$$

where w is the filter bandwidth and n is the filter order.

Step 3: After filtering, the filter is normalized to its power spectrum (PN). The power-normalization expression is as follows:

(7) $$U_{i}=\frac{{Pow}_{n}(\omega_{g})}{\mu [\omega ]}$$

where ${POW}_{n}$ is the value obtained from the Gammatone filter and μ[ω] is the average power.

Step 4: Power function nonlinear processing is expressed as follows:

(8) $${POW}_{n}=U_{n}^{\vartheta }$$

where ϑ is the exponential factor, while in general 0<ϑ<1.

Step 5: The CEPNCC eigencoefficients are the obtained eigenmatrices.

After obtaining the CEPNCC feature parameters through a series of processing steps, the CEPNCC feature parameters are mixed with the time domain features and input into the neural network for training.

### 2.5. Net Modeling

BiLSTM was utilized for processing PPG recordings in the development of AF classification models in this study. BiLSTM, comprising a forward LSTM and a backward LSTM, is well-suited for modeling time series data and is commonly employed in natural language processing tasks to capture bidirectional temporal dependencies and learn contextual information.

**Figure 2:**
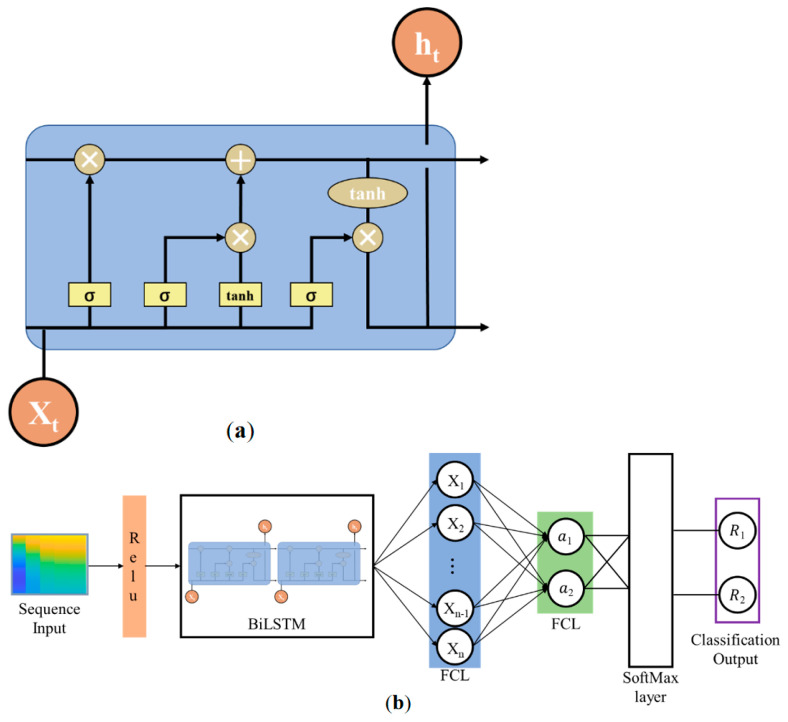
BiLSTM and classification network. (**a**) Basic unit of BiLSTM; (**b**) Block diagram of classification network: The input sequence is activated by ReLU and passed into BiLSTM to generate sequence features (X_1_, X_2_, …, X_n_). These features are extracted through the fully connected layer (FCL) to produce high-level features (a_1_, a_2_), which are then converted to probability distributions by the SoftMax layer, and the final output is the classification result (R_1_, R_2_).

In this study, a neural network model based on BiLSTM was constructed for AF classification. Figure 2a illustrates the basic unit of the BiLSTM, while Figure 2b depicts a block diagram of the overall model. The CEPNCC feature matrix obtained from PPG recordings serves as the input for calculations in this model.

The BiLSTM model (Figure 2) was utilized for binary classification. The PPG recordings diagnosed as AF were grouped into one category, while non-AF rhythms were categorized separately.

To comprehensively characterize the PPG record features, the periodic time domain and frequency domain features were first normalized and activated before being input into the BiLSTM network for training. Subsequently, the classification of PPG recordings was achieved using a SoftMax classifier, which is well-suited to the specific classification requirements of this paper. The SoftMax classifier transforms the logits from the BiLSTM model into a probability distribution by applying the SoftMax function. This allows the score for each category to be interpreted as the probability of that category. Notably, the SoftMax classifier is characterized by its computational simplicity and high efficiency. The number of categories corresponds to clinical diagnostic categories associated with the PPG recordings. Specifically, the presence or absence of AF is indicated by [1] and [0], respectively.

## 3. Experimental Results

After conducting a comprehensive analysis of data preprocessing techniques, the subsequent crucial step involves extracting relevant features from the preprocessed signals to facilitate the accurate classification of AF.

### 3.1. PPG Recordings

**Figure 3:**
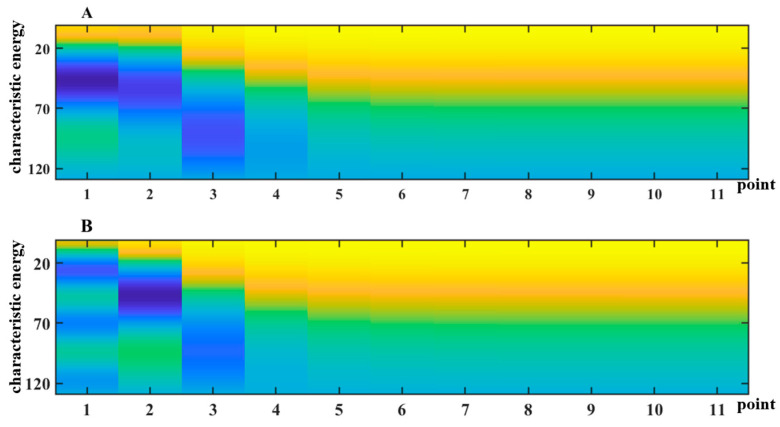
Characteristic Matrix Obtained by CEPNCC. (**A**) AF patient (**B**) Sinus rhythm.

In this study, we identified peak points in the database of PPG recordings and subsequently grouped and segmented them. The sampling frequency of the PPG recordings from the MIMIC-III database was 125 Hz. Therefore, 15,000 data points starting from the peak were selected as the data sample for this experiment. We obtained the feature matrix from the CEPNCC decomposition, as shown in Figure 3. Due to the intermittent fluctuations in the PPG recordings of AF patients, their feature matrix exhibited confounding patterns in the low-frequency domain, with a more concentrated energy distribution in the image. As illustrated in Figure 3, the NSR feature matrix and the AF feature matrix displayed significant differences in amplitude and distribution. The AF feature matrix showed higher energy and uneven distribution at the initial points, with more low-value regions, indicating greater energy variations in AF patients. In contrast, the NSR feature matrix had a more uniform energy distribution and exhibited smaller fluctuations, reflecting the more stable energy variations in patients with normal sinus rhythm. The BiLSTM network captures these features effectively. After combining the frequency domain feature matrix with the time domain features, we employed the BiLSTM network for training.

### 3.2. Net Training and Analysis

To ensure optimal performance of the neural network, it is crucial to carefully set and fine-tune the network parameters. This includes selecting an appropriate optimizer and determining the optimal learning rate, as these factors significantly influence the accuracy and efficiency of the AF detection model.

#### 3.2.1. Network Settings

**Figure 4:**
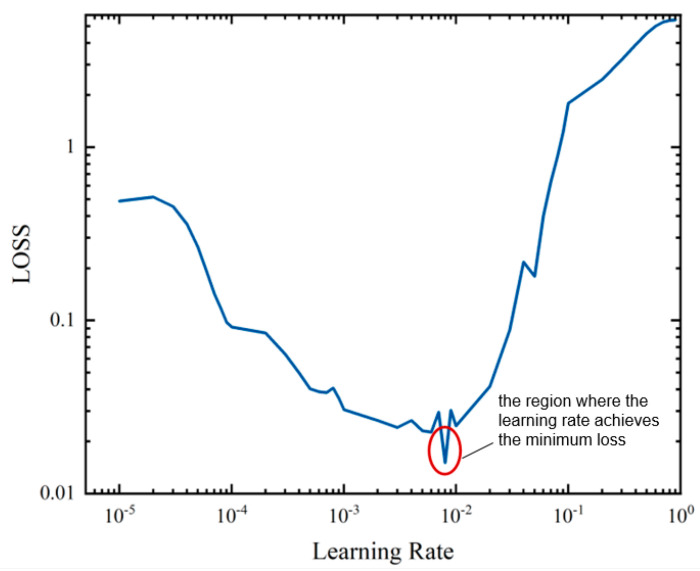
Relationship between Different Learning Rates and Loss.

We set the network structure parameters according to the description in the previous Section 2.5. Following the testing of different network parameters, the Adam optimizer was chosen to regulate the learning rate, which could significantly impact the final AF detection results in machine learning. To select an appropriate learning rate, we designed an optimal learning rate estimation method. A very small initial learning rate for the AF detection network was chosen in this work. The learning rate was increased after each batch, with the retention of the loss obtained from each batch. Subsequently, we created a plot of the learning rate versus loss curve, as shown in Figure 4.

As illustrated in Figure 4, the learning rate achieving the minimum loss was located roughly within the interval [${0.6\times 10}^{-2},{0.2\times 10}^{-1}]$. To accurately determine the optimal learning rate, the accuracy rate α should satisfy the following equation:

(9) $$\alpha =f\left(\varphi ,\omega \right)$$

where φ is the network loss rate and ω is the initial learning rate. The optimal estimate of the network’s initial learning rate is determined by the following equation based on the obtained accuracy parameters $\left(\varphi_{i},\alpha_{i}\right)$:

(10) $$L\left(\alpha ,f\left(\varphi ,\omega \right)\right)=\sum_{i=1}^{N}{\left[\alpha_{i},-f(\varphi_{i},\alpha_{i})\right]}^{2}$$

**Figure 5:**
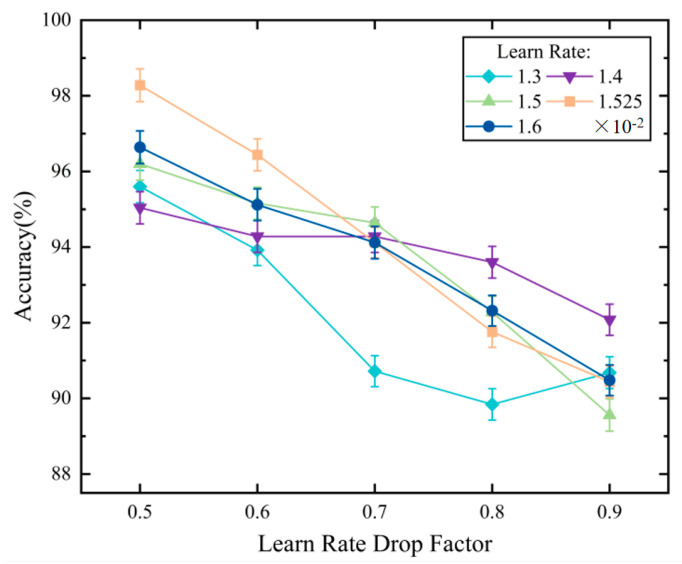
Relationship between Different Learning Rates and Learning Rate Drop Factors and Accuracy.

The minimum value can be obtained from the sequence $\left[\omega_{1},\omega_{2},\ldots ,\omega_{i}\right]$. The relationship between various learning rates, learning rate decay factors (LRDFs), and the network’s classification accuracy was analyzed to select the appropriate LRDF. The results are presented in Figure 5.

It should be noted that both different learning rates and LRDFs can affect the accuracy of the network. As the LRDF increases, there is generally a decrease in the classification accuracy for different learning rates; notably, when the LRDF is 0.5, the classification accuracy reaches its peak. The moderate learning rate decay allows the model to gradually converge during training while maintaining adequate exploration capabilities. This ultimately enables the model to effectively find the optimal solution. When the LRDF falls below 0.5, the AF classification accuracy drops below 80%. This slow decay leads to inadequate parameter adjustment during training and suboptimal performance with decreased classification accuracy. In order to ensure the clarity of the image, the accuracy of the network with an LRDF less than 0.5 is not listed in Figure 5. Therefore, the initial learning rate used for our network was 0.01525, the LRDF was 0.5, and the maximum iteration period was 100.

#### 3.2.2. PPG Classification Recognition Rate of CEPNCC-BiLSTM

**Figure 6:**
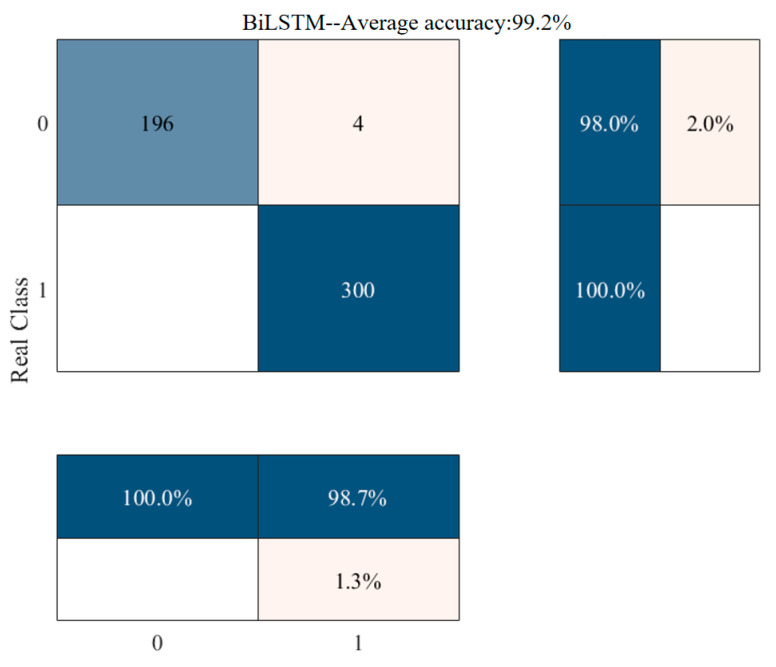
Multi-term, CEPNCC AF Classification Confusion Matrix.

We utilized the CEPNCC feature matrix, extracted from the PPG recordings, for training, classifying, and validating the network. The categorical data in Table 4 were derived from the confusion matrix presented in Figure 6. The model acquired through network training was cross-validated on the test dataset, yielding a final classification accuracy of 99.20%. As depicted in Table 4, the proposed method demonstrates the precise detection of PPG recordings in AF patients with a recall rate of 100%. For PPG recordings from NSR, the false alarm rate was merely 2%. Across multiple experiments, CEPNCC-BiLSTM consistently exhibited the accurate screening of AF patients.

#### 3.2.3. Compared with Existing Methods

To demonstrate the superiority of the proposed method in AF classification, we compared it with other methods, including EPNCC-BiLSTM, CEPNCC-BiLSTM, SVM, EPNCC-SVM, and CEPNCC-SVM. All the models were trained on the same hardware environment using the same training set and test set.

As illustrated in Table 5 and Table 6, the SVM classifier was capable of producing results in a relatively short time; however, it exhibited a significant loss of accuracy and an AF false alarm rate of 26%, which was insufficient to meet the requirements for AF detection. In contrast, AF detection could be classified rapidly and with minimal loss of accuracy by employing EPNCC or CEPNCC to transform PPG recordings into feature matrices. Nevertheless, it should be noted that there were notable differences between the aforementioned two methods for AF detection. EPNCC required a considerable number of calculations to fulfill the accuracy requirements due to residual errors, which consequently prolonged its preprocessing time.

The application of CEPNCC for the preprocessing of PPG recordings had the effect of reducing both the necessary preprocessing and training times while simultaneously achieving a high degree of accuracy. As illustrated in Table 5, the CEPNCC-BiLSTM classification method exhibited high sensitivity in AF detection and was capable of accurately classifying PPG recordings with AF features. In multiple experiments, the proposed method demonstrated the capacity to not only detect AF features in PPG recordings with precision but also achieve a markedly lower false-positive rate than other methods under comparison.

#### 3.2.4. Proposed Evaluation Methodology

Table 7 demonstrates that the EPNCC-BiLSTM achieved a classification accuracy of 91.46% and a recognition accuracy of 97.79%. However, it exhibited the slowest computation speed, resulting in the lowest *ET-score*. Conversely, using only SVM as a classifier yields faster computation but lower *ET-score* due to decreased accuracy. When EPNCC-SVM was employed for AF classification, it attained an accurate AF recognition rate of 93.62% with rapid computational speed. In contrast, CEPNCC-SVM proved relatively more efficient with an AF detection accuracy of 96.18%. Notably, CEPNCC-BiLSTM outperformed all the methods by achieving the highest *ET-score* through preprocessing PPG recordings in approximately 5.8 s and achieving exceptional AF detection accuracy at 99.2%. Furthermore, CEPNCC-BiLSTM consistently demonstrated high sensitivity to AF detection across multiple experiments, with most false positives originating from PPG recordings of non-AF patients.

## 4. Discussion

This study proposes an intelligent method for AF detection based on long-term photoplethysmography (PPG) data and the CEPNCC-BiLSTM model. The experimental results demonstrate that this method significantly outperforms existing approaches in terms of accuracy and efficiency, addressing the issue of under-detection caused by the AF’s paroxysmal nature and meeting AF detection requirements.

The CEPNCC-BiLSTM model enhances AF detection sensitivity and specificity by combining CEEMD and PNCC techniques to extract both time domain and frequency domain features. CEEMD efficiently processes non-smooth signals, contributing to reduced computation time and improved accuracy. Additionally, the bidirectional characteristic of the BiLSTM model enables better capture of contextual information in time series data, enhancing its ability to recognize AF features.

Compared to traditional methods, the CEPNCC-BiLSTM model excels in various performance metrics. When compared to EPNCC-BiLSTM, our proposed method not only improves the detection accuracy (99.2% vs. 93.62%) but also significantly reduces the preprocessing time (5.8291 s vs. 36.1267 s). In comparison with the SVM method, the CEPNCC-BiLSTM model demonstrates superior accuracy (99.2% vs. 96.18%) and computational efficiency. Moreover, these improvements are attributed to the efficient preprocessing by CEEMD and the robust time series processing capability of the BiLSTM model, which reduces the computational overhead while capturing signal features effectively. Furthermore, the CEPNCC-BiLSTM model exhibits high efficiency in processing non-smooth signals, making it more stable and reliable in long-term monitoring: a crucial characteristic for practical applications.

The proposed method holds great potential for practical applications, particularly in long-term AF monitoring. Its efficient processing capabilities make the CEPNCC-BiLSTM more suitable for wearable devices and real-time monitoring systems, which can help improve early detection rates of AF, thereby reducing the risk of stroke and other complications without enabling continuous cardiac health monitoring.

While the CEPNCC-BiLSTM method demonstrated strong performance, it also exhibited certain limitations. Firstly, the diversity and size of the dataset may impact the model’s ability to generalize. This study utilized publicly available data, consisting of 400 healthy human PPG recordings from the Synthetic Dataset and 1100 PPG recordings of AF patients from the MIMIC-III Waveform Database. To improve the model’s generalization performance, additional AF data were appropriately selected. The MIMIC-III Waveform Database was obtained from bedside monitors in an intensive care unit (ICU) within an inpatient unit. Among the AF PPG recordings, 89.09% of patients had a sole diagnosis of AF, while another 10.91% had a concurrent diagnosis of AF and other disorders; however, detailed information regarding these disorders was not disclosed by the data organization. ICU patients typically exhibit more severe physiological states due to high stress levels and various comorbidities, which can introduce complexity into PPG recordings. Consequently, these data might not accurately represent outpatient or community patient populations, thereby limiting their applicability to other scenarios such as general wards or outpatient clinics.

The experimental results in Figure 6 revealed four false positive samples, which can be attributed to (1) significant individual differences among samples and (2) interference from other diseases affecting the model’s learning of atrial fibrillation-related features. Consequently, enhancing the model’s generalization ability will be a primary focus in our team’s future research endeavors. Furthermore, incorporating more diverse population data and exploring atrial fibrillation detection effectiveness across different contexts are essential considerations. Additionally, future research should encompass classifying various arrhythmias (e.g., ventricular premature beats and tachycardia) to offer a comprehensive cardiovascular health monitoring solution while identifying other sources of interference in arrhythmia signals.

## 5. Conclusions

In this study, an intelligent method named CEPNCC-BiLSTM has been proposed for the detection and diagnosis of AF with long-term PPG recordings. This method significantly improves the accuracy of AF detection and overcomes the limitations of intermittent monitoring in traditional methods. Additionally, it achieves superior classification accuracy while demonstrating improved computational efficiency compared to other long-term monitoring methods. Specifically, our approach accurately identifies PPG signals with AF characteristics, achieving an impressive accuracy rate of 99.2%. Moreover, it processes 2 min PPG recordings in just 5.8 s, making it the fastest among the five methods considered in this study. Furthermore, we introduce a novel performance metric based on the *ET-score* that incorporates a temporal dimension beyond F-measure evaluation. The *ET-score* for CEPNCC-BiLSTM is evaluated to be the highest among all five methods, further confirming its effectiveness. Importantly, our research findings demonstrate that PPG signals contain sufficient information for precise and rapid differentiation between AF and normal NSR using CEPNCC-BiLSTM. Therefore, long-term PPG recordings can be effectively used for accurate AF detection.

## Figures and Tables

**Table 1:** Diagnosis Distributions in the Training and Test Sets.

Signal Category	Training Set	Test Set
PPG from AF patients	75%	46%
PPG from AF patients with other diseases	5%	14%
Sinus rhythm PPG	20%	40%

**Table 2:** Feature Extraction Time and Network Training Time.

Data	Feature Extraction Time (Per Data)	Network Training Time	Average Accuracy (%)	*ET-Score*
1 min PPG	About 3 s	1 min 16 s (Only 400 data items)	96%	96.25
2 min PPG	About 5 s		98%	96.80
5 min PPG	About 10 s		98%	92.50

**Table 3:** Processing of Two Methods.

Method	Required Processing Times (Times)	Average Processing Time (s)
CEEMD	20	5.8291
EEMD	200	36.1267

**Table 4:** CEPNCC-BiLSTM Classification Recognition Rate.

Category	Recall Rate (%)	Precision (%)
AF Patient	100	98.7
Sinus rhythm	98	100

**Table 5:** Recall Rate of Each Training Category.

Category	EPNCC-BiLSTM (%)		CEPNCC-BiLSTM (%)		SVM (%)		EPNCC-SVM (%)		CEPNCC-SVM (%)	
PPG recordings	AF	NSR	AF	NSR	AF	NSR	AF	NSR	AF	NSR
Recall rate	93.5	100	98.7	100	74	100	88.8	100	92.9	99.4
Precision	89.5	95.67	100	98	100	71.9	100	81	99.7	88.5

**Table 6:** Training Time and Preprocessing Time of Each Training Category.

Average Time (1500-Data)	EPNCC-BiLSTM	CEPNCC-BiLSTM	Input PPG Recordings to SVM	EPNCC-SVM	CEPNCC-SVM
Preprocessing	15 h	2.25 h	0	15 h	2.25 h
Training	3 min 16 s	3 min 10 s	1 min 7 s	About 5 s	About 5 s

**Table 7:** F-measure and *ET-score* of each training category.

Category		EPNCC-BiLSTM	CEPNCC-BiLSTM	SVM	EPNCC-SVM	CEPNCC-SVM
F-measure	AF Patient	91.46%	100%	85.06%	93.62%	96.18%
	Sinus rhythm	97.79%	98.34%	83.65%	89.50%	93.63%
*ET-score*		82.28	97.27	84.35	86.10	92.99

## Data Availability

The PPG dataset adopted in this research is openly available in [Physionet] at the MIMIC-III Waveform Database v1.0 (physionet.org) and https://archive.physionet.org/cgi-bin/atm/ATM (accessed on 7 June 2024).
